# Learning the layout of different environments: common or dissociated abilities?

**DOI:** 10.1186/s41235-025-00618-5

**Published:** 2025-02-21

**Authors:** Alexis Topete, Chuanxiuyue He, Mary Hegarty

**Affiliations:** Department of Psychological and Brain Sciences, University of California, Santa Barbara, USA

**Keywords:** Spatial ability, Navigation, Virtual environments, Confirmatory factor analysis

## Abstract

People navigate in various types of spaces, including indoor and outdoor environments. These differ in availability of navigational cues, such as distal landmarks, clear boundaries, and regular grid structures. Does learning the layout of different types of environments rely on the same or diverse cognitive abilities? Do separate measures of learning reflect different abilities? In a study of individual differences, 88 people learned the layout of two virtual environments from first person experience: a grid-like maze, and a campus-like open environment. After learning each environment, their knowledge was measured by three tasks; onsite pointing, map-reconstruction, and wayfinding. Performance on these measures was significantly correlated. In confirmatory factor analyses, the best fitting model indicated separate factors for spatial knowledge acquisition of the grid-like maze and the outdoor open environment. However, these two factors also shared considerable variance, indicating that they reflect a common underlying ability. There was no evidence that different measures of learning (pointing, map reconstruction, and wayfinding) defined separate abilities, adding to their validity as alternative measures of configural knowledge. Performance of map-based navigation and path integration in the mobile navigation game Sea Hero Quest was generally not correlated with performance in the environment learning tasks, nor were self-report measures of sense of direction and spatial anxiety. Our research suggests that there is a common ability related to learning spatial layout in different contexts, but this may be distinct from other navigation abilities.

**Supplementary Information:**

The online version contains supplementary material available at 10.1186/s41235-025-00618-5.

## Significance statement

The ability to navigate in different types of spaces, including neighborhoods and buildings, is required in everyday life, yet we know little about how the structures and scales of different environments affect navigation proficiency. Does the ability to learn a large, irregular, outdoor environment depend on the same or different cognitive processes as the ability to learn a small, grid-like, indoor one? Additionally, does it matter how we measure navigation ability after learning a spatial layout? These questions have important implications for theories of how people differ in navigation ability, and for the measurement of navigation for real-world applications such as the detection and diagnosis of Alzheimer’s Disease, and training for professions that are navigation-intensive, such as emergency responders. We studied navigation in different contexts examining how people learn the spatial layout of two different types of environments (large, outdoor, and irregular vs. small, indoor, and grid-like) and assessed their resulting knowledge of these environments using three measures; ability to point to landmarks, ability to reconstruct a map of the environment, and ability to find their way efficiently in the environment. Results indicated that the three outcome measures seem to reflect a single navigation ability, and there is some dissociation between learning indoor and outdoor environments, although these also depend to a large extent on a common ability.

## Introduction

Navigation is an essential component of daily life. Whether it is finding your way from your office to the restroom in a large building or finding a meeting in a building across a university campus, having knowledge of the layout of environments is essential to planning and executing efficient routes to goal locations. Our ability to learn the layout of novel environments from experience navigating in these spaces is remarkable, but also subject to large individual differences (Wolbers & Hegarty, 2010). These individual differences are now well documented (e.g., Allen et al., 1996; Hegarty et al., 2006; Ishikawa & Montello, 2006; Weisberg & Newcombe, 2018), raising important theoretical questions about what causes these differences, what cognitive processes differentiate more and less able navigators, and whether navigation abilities can be trained.

To address these issues, we need standardized measures of navigation ability. However, to date, studies of this ability have been conducted in different spaces (e.g., grid-like buildings vs. open, rural environments) and used a variety of tasks and measures, and we know little about the relationships between existing tasks and measures. Here, we begin to address this problem by assessing a navigation task in two different virtual environments via the same three outcome measures for each, and examining relations between these measures.

Navigation ability includes the ability to learn the layout of new environments, to update our position in space as we navigate, and to use our knowledge of known environments plan and execute efficient paths to goal locations (also referred to as wayfinding). As such, there are many tasks that can and have been used to measure this ability. In this paper we focus on one common paradigm used to measure navigation ability in the literature to date. This paradigm includes a *learning phase,* in which people are given first-person experience navigating a novel environment, followed by a *test phase* to measure their *environmental learning*, or how well people learned the layout of the environment from this direct experience. Typically, researchers control the learning phase by having people follow a guided route (or series of guided routes).^1^ Participants are then tested on their configural (or survey) knowledge of the environment via one or more tasks, with the assumption that good navigators can go beyond memorizing the sequence of viewpoints experienced during the learning experience (i.e., a route representation, which relies more on knowledge of egocentric relations between the self and environmental features experienced during learning) to infer a representation of the layout of the environment (Hegarty et al., 2006). This survey/configural representation includes allocentric relations between locations in the environment (i.e., knowledge of the distance and directions between landmarks, independently of how they were experienced during learning) enabling the navigator to point accurately from one location to another and to plan and execute efficient routes (i.e., shortcuts) not experienced during the learning phase (efficient wayfinding).

Table 1 summarizes representative papers that have used this general method. These studies have been conducted in real environments (e.g., Boone et al., 2018; Hegarty et al., 2006; Ishikawa & Montello, 2006; Meneghetti et al., 2021; Miola et al., 2021; Montello & Pick, 1993; Schinazi et al., 2013), immersive virtual environments (e.g., Chrastil & Warren, 2015; He et al., 2023) and increasingly in desktop virtual environments (e.g., Münzer et al., 2020; Weisberg et al., 2014).

**Table 1:** Examples of previous research examining navigation performance after learning via a guided route or series of routes in the environment, with key characteristics defined, listed in chronological order

Study	Environment learned	Measures of learning
Montello and Pick (1993)	Indoor Building (Real)	Onsite pointing (arrow circle) Sketch mapping
Hegarty et al. (2006)	Indoor Building (Real) Indoor Building (Video) Indoor Maze (Virtual)	Onsite pointing (arrow circle) Distance estimation Map drawing
Ishikawa and Montello (2006)	Outdoor Wooded Neighborhood (Real)	Onsite pointing Distance estimation Map drawing
Schinazi et al. (2013)	Outdoor Campus (Real)	Onsite and offsite pointing Distance estimation Sketch mapping
Weisberg et al. (2014); Weisberg and Newcombe (2016)	Outdoor Campus (Virtual)	Onsite pointing Map reconstruction
Hejtmánek et al. (2018)	Outdoor City (Virtual)	Wayfinding efficiency Onsite pointing
Ishikawa (2019)	Outdoor City (Real)	Route tracing Onsite pointing (paper)
Münzer et al. (2020)	Outdoor Village (Virtual)	Wayfinding accuracy & efficiency Onsite pointing Map reconstruction
Meneghetti et al. (2021)	Garden Maze (Virtual)	Route tracing Navigation efficiency Map completion
Miola et al. (2021)	Outdoor City (Virtual)	Offsite pointing Map completion
He et al. (2023)	Indoor Maze (Virtual)	Wayfinding efficiency Offsite pointing
Muffato et al. (2023)	Outdoor City (Video)	Landmark knowledge Offsite pointing Route tracing Sketch mapping

The research studies listed in Table 1 are representative studies only and do not encompass all research studies using the discussed paradigm

The types of environments that people learned in these studies have also differed. They include outdoor, or simulations of outdoor environments, such as a campus, city, or natural environment (e.g., Gagnon et al., 2018; Ishikawa & Montello, 2006; Miola et al., 2021; Muffato et al., 2023; Weisberg et al., 2014), and smaller built environments (or simulations of these environments), such as a building or maze (e.g., He, et al, 2023; Hegarty et al., 2006; Meneghetti et al., 2021). Although all of these qualify as environmental scale environments, according to Montello’s (1993) classification of scales of space, they differ in size, and therefore the amount of physical effort or time required to traverse the area. They also differ in regularity and in number and size of possible turn angles—indoor grid-like environments typically have primarily 90-degree angles and straight paths; whereas outdoor environments can be more irregular, with curved paths, a range of turning angles, and differ in the availability of distal landmarks (such as distinctive buildings, mountains, and the sun).

Additionally, the studies summarized in Table 1 differ in the tasks used to measure the quality of configural or survey knowledge acquired from the learning experience. These include ability to point directly from one location and orientation in the environment to other locations, including measures of onsite pointing (from within the environment) and measures of offsite pointing (e.g., *Judgments of Relative Direction),* that is, from an imagined location and orientation in the environment. They also include *sketch mapping* (drawing a map of the environment) or *map reconstruction* (recreating a map of the environment by moving icons representing landmarks to indicate their relative positions), straight-line *distance estimations* between locations in the environment. Finally, they include efficient *wayfinding* (ability to plan and execute shortcuts, or efficient paths between landmarks that were not experienced during the learning phase). While some studies also include measures of landmark knowledge and route knowledge, here, we focus on measures of configural knowledge, which involves the ability to infer the layout of an environment from route experience. Moreover, the present study is concerned with measures of ability, and not navigation strategy (e.g., Boone et al., 2018; Marchette et al., 2011).

Despite differences in environmental structure and outcome measures, it is implicitly assumed that the same environmental learning ability is reflected in all outcome measures and for all environments. This is characterized as the ability to construct a configural or survey representation of the environment, from direct experience (Montello, 2005; Tolman, 1948), with better navigators developing more globally consistent and metrically accurate representations.

However, there are reasons to believe that there might be variation in the abilities necessary to learn the layout of different environments and to perform different outcome measures. With respect to the type of environment, people who grew up in more irregular environments (e.g., rural environments vs. cities with high street entropy) have been found to have superior ability to those who grew up in grid-like cities, and people generally navigate more efficiently in the types of environments they grew up in (Barhorst-Cates et al., 2021; Coutrot et al., 2022). These results suggest that people may develop different navigation abilities depending on their experiences and that learning of different types of environments may draw on distinct abilities. For example, someone who pays more attention to distal landmarks might have an advantage in learning outdoor environments with these cues, but not indoor environments.

With respect to outcome measure, it is argued that rather than metrically accurate survey representations, spatial representations such as *graph knowledge* may be sufficient to perform well on some outcome measures but not others. Graph knowledge comprises topological spatial information that represents locations within an environment as nodes, connected by paths or edges (Kuipers, 1982, 2000), but does not include accurate metric knowledge of distances and directions among locations. Chrastil and Warren (2013, 2015; Warren, 2019) have argued that graph knowledge (i.e., which landmarks are connected by paths) is sufficient for tasks such as efficient wayfinding, as this type of representation can enable the inference of novel routes. In contrast, other tasks such as pointing may require more globally consistent metric knowledge, for example of the relative length of the different paths, and the direction of the paths with respect to some global orientation. In support of this distinction, when both wayfinding and pointing are measured in the same studies, correlations between these tasks are not always high and, in consequence, could reflect different types of knowledge (e.g., He et al., 2023; Muryy & Glennerster, 2021). Moreover, a recent study also suggested that the type of knowledge constructed depends on the type of environment, with representations of more open environments showing properties of metric survey knowledge, whereas representations of more maze-like environments showed properties of graph knowledge (Peer et al., 2024). These studies raise the possibility that different abilities may be associated with different environments and outcome measures.

Alternatively, a common ability may underlie learning of different environments and measure of environments, such that people vary in the amount of metric accuracy and precision of their representations following a learning experience (Peer et al., 2021). Because studies rarely measure learning of more than one environment (Peer et al., 2024), and learning outcomes vary among studies, we do not know the relations between tasks that use different environment types or different measures of learning. In the present study, we begin to fill this gap in our knowledge by addressing two primary questions: (1) Does learning of *different environments (i.e. grid-like indoor environments and irregular outdoor environments)*, involve the same or different navigation abilities? (2) Given the *same environment*, do different measures of environment layout (i.e., onsite pointing, map reconstruction and wayfinding efficiency) measure the same or different abilities?

We focus on tasks using desktop virtual environments as these have the potential to be standardized measures of navigation ability that can be used by researchers across the world to study learning of the same environment (in contrast to real environments which are different everywhere, and ambulatory immersive environments which require large laboratories and expensive equipment). Using VR allows researchers to control for external factors such as weather, cue availability, size, and environmental structure and there is increasing evidence that navigation abilities measured in virtual environment are reflective of real-world navigation (e.g., Claessen et al., 2016; Coutrot et al., 2018; Lader et al., 2024; van der Ham et al., 2015).

To address these questions, we had people learn the layout of two different virtual environments, and in each case measured their knowledge of the layout of these environments using the same three outcome measures (efficient wayfinding, pointing, and map reconstruction). The first environment was Virtual SILCton (Weisberg & Newcombe, 2016; Weisberg et al., 2014), which simulates a college campus (an open irregular environment). In the SILCton task, participants are led on two separate routes in the environment, before experiencing two connecting routes and their knowledge is tested by pointing and map-reconstruction measures. In the present study we added efficient wayfinding as a third outcome measure. The second environment simulates an indoor, grid-like maze environment. This virtual environment was first used by Marchette et al (2011) to measure navigation strategy so we call it the Marchette Maze. Here we modified the task to measure wayfinding *ability*, by instructing participants to find the shortest route to goal locations (cf. Boone et al., 2019) and added onsite pointing and map reconstruction outcome measures. We chose these types of environments because they are quite different from each other, which enabled a strong test of whether ability to learn the layout of diverse environments depends on the same or different cognitive abilities, and also because they are types of environments that the average person tends to navigate in on a regular basis, and which have been used in previous studies of individual differences in large-scale spatial ability.

The main aim of the current study was to evaluate three different models of navigation ability using confirmatory factor analysis. The first model assumes that the six outcome navigation measures reflect a single “environmental learning” or navigation ability (Montello, 2005; Peer et al., 2021). The second assumes that there are separate abilities associated with learning different types of environments (a large, open, outdoor environment such as Virtual SILCton, and a small, indoor, grid-like environment such as the Marchette Maze) (Barhorst-Cates et al., 2021; Coutrot et al., 2022; Peer et al., 2024). The third assumes that there are three separate abilities associated with the outcome measures (i.e., wayfinding, pointing, and map reconstruction) (Chrastil & Warren, 2013, 2015; He et al., 2023; Muryy & Glennerster, 2021; Peer et al., 2024; Warren, 2019).

While ability to learn the layout of a novel environment is a dominant method of assessing navigation ability, there are other common standardized measures of this ability, including self-report measures (Hegarty et al., 2002; Lawton, 1994) and more recently, Sea Hero Quest (Coutrot et al., 2018), a mobile video game that measures two abilities; *map-based navigation*, the ability to navigate to a series of landmarks (buoys in a body of water) after memorizing a map of the environment) and *path integration* (ability to point back to the start location after traveling in an environment) and which has been found to be predictive of real-world navigation ability (Coutrot et al., 2019; Spiers et al., 2021). There is concern that navigation in desktop virtual environments is affected by interface facility provided by video-game experience (Hegarty et al., 2022; Yavuz et al., 2024). To advance our understanding of individual differences in navigation ability, it is also important to examine whether self-reports and different navigation tasks reflect the same ability as learning the layout of new spaces and how these might be affected by video game experience. Consequently, we also included these measures and examined their correlations with measures of learning spatial layout in exploratory analyses, to begin to study broader questions regarding the measurement of navigation ability.

## Methods

### Participants

Participants were undergraduate students recruited through the institution’s participant pool, with 111 recruited to achieve 88 participants in the final dataset. Of these participants, 21 were dropped due to motion sickness, not returning for the second session, or technical errors with one or more tasks. Two additional participants were dropped because they were identified as multivariate outliers measured by Mahalanobis’ Distance (Curran, 2016; Ghorbani, 2019; Ward & Meade, 2022), based on all objective measures. Participants in the final sample of 88 ranged from 18 to 30 years of age (*M* = 19.92, *SD* = 2.46, 52 female, 36 male), and received either cash ($40) or course credit (3 h of subject pool credit) in exchange for participating.

An a priori power analysis was conducted using the semPower package in R (Moshagen & Bader, 2023) to estimate the required sample size for detecting differences between one-factor, two-factor, and three-factor models. Based on model-implied covariance matrices, the analysis indicated that at least 85 participants are needed to achieve 80% power at an alpha level of 0.05 (see details about the assumed parameters in the shared supplementary codes). This sample size also gave us power to detect correlations of 0.28 or higher, assuming power of 0.80 and an alpha level of 0.05.

### Materials

The measures were completed either on a desktop computer with a keyboard and mouse, or (in the case of Sea Hero Quest) via a touch-screen phone that was provided by the lab. The following measures were included in the study (see Figure S1 in the Supplementary Materials for more images of the Virtual SILCton and Marchette Maze tasks).

#### Virtual SILCton

**Fig. 1:**
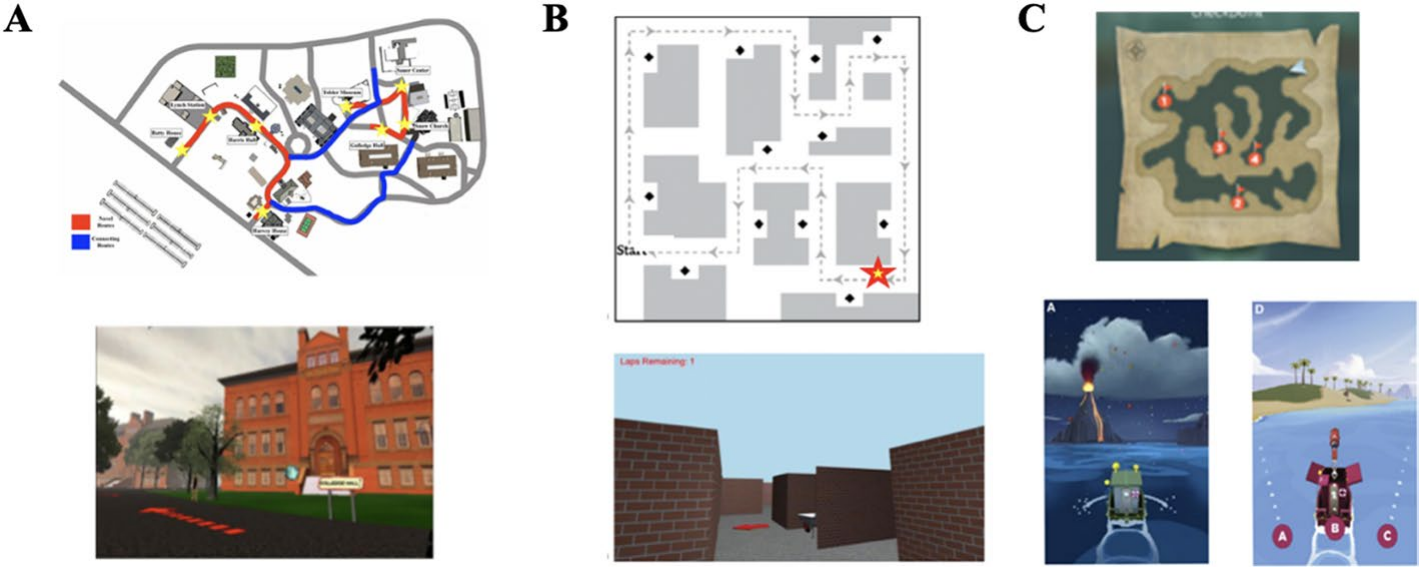
Aerial view images of each environment studied, and a screenshot of one point of view in the environment. Map and first-person view of **A** Virtual SILCton environment. **B** Marchette Maze environment. **C** Sea Hero Quest environment

The Virtual SILCton task (Weisberg et al., 2014) is an assessment of navigation ability administered via a desktop computer, keyboard, and mouse, and is a virtual simulation of Temple University’s Ambler campus which is approximately 500 × 500 m squared. Participants are tasked with memorizing the names and locations of 8 buildings within a large, campus-like environment by following a series of guided routes in the learning phase (see Fig. 1A). They first learn two separate routes, each of which contains four unique landmarks (buildings), by traversing them in the forward and backward direction, and then learn two routes that connect these, but which do not contain any additional landmarks.

The measures of learning included an onsite pointing task, a wayfinding task and a map-reconstruction task. In the onsite pointing task, participants are placed in front of each of the 8 learned landmarks, in turn, and are asked to point to each of the other 7 landmarks in a random order (56 pointing trials). Of these, 24 are within-route trials that involve pointing to landmarks within the two originally learned routes and 32 are between-route trials that involve pointing between these routes. On each trial participants respond with a mouse click after using the mouse to rotate their viewing direction on a horizontal plane to their judged direction of the landmark. The measure of performance on each trial is the absolute angular deviation of the target building’s actual direction and the participant’s judged direction, averaged separately for within-route trials and between-route trials, such that greater angular error is associated with poorer performance.

In map reconstruction, participants are given a blank map of the environment and are instructed to use the mouse to drag-and-drop pictures of the 8 learned buildings into their correct locations within a map. There is no time limit. The measure of performance is based on a bidimensional regression analysis (Friedman & Kohler, 2003) which measures the variance shared between the participant’s placement of the 8 landmark buildings and the actual locations in X, Y coordinates of these buildings.

On each trial of the efficient wayfinding task, participants were placed in front of one of the previously learned buildings in the SILCton environment, and were instructed to take the shortest possible path to one of the other 7 buildings. They were allowed to take any route (e.g., they could walk across a lawn or parking lot), but could not walk through any buildings. They used a mouse and keyboard interface to move through the environment and complete a total of 8 trials in the wayfinding task; 4 within-route trials (two from each route) and 4 between-route trials. Participants were allowed 6 min on each trial with the exception of 2 within-route trials in which they were allowed 3 min (based on pilot testing). The end point of each wayfinding trial was the starting point for the next trial and if participants exceeded the time limit for a trial, they were scored as unsuccessful on that trial and a researcher moved them to the starting point for the next trial. The measure of wayfinding efficiency was excess distance, or the difference between the actual distance a participant traveled (*d*_*A*_) and the shortest possible distance (*d*_*S*_), divided by the shortest possible distance (*d*_*S*_) so that larger numbers indicate less efficient wayfinding:

$$efficiency\;(excess\;distance) = \frac{dA - dS}{{dS}}$$

#### The Marchette Maze task

The Marchette Maze simulated a smaller (55 × 55 m, Boone et al., 2018), indoor maze-like environment (see Fig. 1B), so named because it had the same structure as the maze used in studies of the Dual Solution Paradigm (Marchette et al., 2011). In the learning phase, participants followed a single guided route (indicated by arrows) that passed 12 landmark objects: a trash can, bookshelf, wheelbarrow, harp, well, chair, mailbox, telescope, plant, picnic table, stove, and piano. They completed 5 laps of the route. On the first lap, they name each object aloud, and the experimenter corrects them if they use a name that is different to the name used to refer to that object in subsequent test trials.

The onsite pointing task consisted of 20 trials, similar to the pointing trials in the Virtual SILCton task, in which participants are placed in front of one of the objects and have to indicate the direction to another landmark by rotating the environment until they believe they are facing the target object. In contrast with SILCton there was no crosshair and participants pressed the “Enter” button when they believed they were facing a target object. The map reconstruction task had the same format as the corresponding task for SILCton. The landmarks were represented as boxes that revealed a picture of each object when the participant hovered over the box with the computer mouse. The wayfinding task followed the same format as the Virtual SILCton task except that (1) there were 20 trials, (2) participants were given a limit of 40 s to find a target object (consistent with previous studies using this maze), 3) they were considered successful if they were within one grid square (equivalent to 5 m) of the target object and 4) the trials were presented in a random order.

#### Sea Hero Quest

Participants completed a subset of 13 trials of Sea Hero Quest (Coutrot et al., 2018): 2 practice trials (Levels 1 and 2), 6 wayfinding trials (Levels 6, 8, 11, 26, 32, and 48) and 5 path integration trials (Levels 24, 34, 44, 54, and 59). Each wayfinding trial’s weighted distance is calculated as the distance traveled in a level (*D*_*x*_) divided by the sum of the distance traveled over the practice Levels 1 and 2 (*D*_*1*_ + *D*_*2*_), to control for interface ability:

$$weighted\;distance\;(trial\;x) = \frac{Dx}{{D1 + D2}}$$

As in previous research on this task, wayfinding efficiency is calculated as the average z-scored weighted distance traveled across the wayfinding levels, after controlling for Levels 1 and 2. Path integration ability is scored as the sum of the stars collected across all corresponding trials, where a higher score indicates better ability (for a total possible score range of 5–15).

#### Self-report measures

We included the Santa Barbara Sense of Direction Scale (SBSOD; Hegarty et al., 2002) the Spatial Anxiety Scale (modified from He & Hegarty, 2020; Lawton, 1994), as self-report measures of spatial ability, as well as video game experience (see Supplementary Materials for the questionnaire). Additional measures that were included, but not reported here, are the Growth Mindset in Navigation, Exploration Tendency, GPS Reliance (He & Hegarty, 2020), GPS Usage (Topete et al., 2024), and the Mindful Attention and Awareness (Brown & Ryan, 2003) scales.^2^

### Procedure

The study was conducted in-person in 2 sessions of 1.5 h each, with at least one day but no more than a week between sessions. In Session 1, participants provided consent via Qualtrics and were then directed to the Virtual SILCton task and completed the learning phase, onsite pointing, map reconstruction, and wayfinding in that order. The learning, onsite pointing, and map reconstruction phases were not timed. Then they completed the self-report measures via Qualtrics in a random order, except that the GPS scales were grouped together (the GPS Reliance Scale presented first, followed by the GPS Usage Scale). After completing the self-report measures, participants were given a one-item open-ended attention check (to recall how they responded to a previous scale item), and a one-item seriousness check to confirm whether they took the study seriously; none of the participants failed these checks. Finally, participants answered demographic questions (age, sex, and ethnicity).

In Session 2, participants returned to the lab and completed the Marchette Maze learning phase, onsite pointing, map reconstruction, and wayfinding, in that order. The learning, onsite pointing, and map reconstruction phases were not timed. Finally, participants completed the Sea Hero Quest levels.^3^

## Results

Descriptive statistics for the Virtual SILCton and Marchette Maze tasks are presented in Table 2. Wayfinding success was close to ceiling performance for both environments (as shown by the Skewness scores), as is typical for these tasks, and therefore justifies focusing our analyses on efficiency rather than success for these measures. Skewness and kurtosis for the other measures indicated that they did not depart from normality. Reliabilities (Omega) were generally high (with perhaps the exception of SILCton wayfinding). As in previous studies of the Virtual SILCton task, within-route pointing was more accurate (*M* = 17.11, *SD* = 12.20) than between-route pointing (*M* = 43.27, *SD* = 19.30) and these measures were highly correlated *r*(86) = 0.69, *p* < 0.001, 95% CI [0.57, 0.79]. For the purpose of this individual differences study, we averaged all the pointing trials into one measure, but the results presented here do not differ substantially if we consider within and between pointing as separate measures (see Tables S1 and S2 and Figure S2 in Supplementary Materials). While the study was not designed to measure sex differences, we note four of the six environment measures showed a significant male advantage with effect sizes similar to other studies of sex differences, as reviewed by Nazareth and colleagues (2019; See Table S3 for descriptive statistics and Welch’s t-tests).

**Table 2:** Descriptive Statistics of Performance in the Navigation Tasks for the Virtual SILCton and Marchette Maze Paradigms (N = 88)

Task	M	SD	Min	Max	Omega	Skewness	Kurtosis
SILCton Onsite Pointing	32.06	15.13	8.18	74.60	0.94	0.45	− 0.61
SILCton Map Reconstruction	0.68	0.25	0.06	0.98	0.96	− 0.94	− 0.09
SILCton Wayfinding Success	0.97	0.05	0.88	1.00	0.79	− 1.36	− 0.16
SILCton Wayfinding Efficiency	0.16	0.10	0.02	0.43	0.60	0.71	− 0.55
Maze Onsite Pointing	42.51	18.65	8.80	95.20	0.84	0.25	− 0.41
Maze Map Reconstruction	0.56	0.32	0.03	0.98	0.88	− 0.12	− 1.59
Maze Wayfinding Success	0.93	0.09	0.60	1.00	0.70	− 1.22	0.76
Maze Wayfinding Efficiency	0.66	0.38	0.02	1.39	0.84	− 0.15	− 1.14

### Correlations

As shown in Table 3, all measures of performance for Virtual SILCton were substantially correlated: wayfinding efficiency and pointing, 95% CI [0.32, 0.64], map reconstruction and wayfinding efficiency, 95% CI [− 0.64, − 0.32], and map reconstruction and pointing ability, 95% CI [− 0.74, − 0.48]. Performance between measures for the Marchette Maze were also highly correlated; wayfinding efficiency and pointing, 95% CI [0.71, 0.86], as well as map reconstruction and wayfinding efficiency, 95% CI [− 0.84, − 0.67] and map reconstruction and pointing ability, 95% CI [− 0.82, − 0.63].

**Table 3:** Correlations between the navigation measures in the Virtual SILCton and Marchette Maze tasks

Variable	1	2	3	4	5
1. SILCton Onsite Pointing	–				
2. SILCton Map Reconstruction	− 0.63***	–			
3. SILCton Wayfinding Efficiency	0.50***	− 0.50***	–		
4. Maze Onsite Pointing	0.61***	− 0.43***	0.35***	–	
5. Maze Map Reconstruction	–0.46***	0.30***	− 0.24*	− 0.74***	–
6. Maze Wayfinding Efficiency	0.56***	− 0.45***	0.38***	0.80***	− 0.77***

*p < 0.05, **p < 0.01, ***p < 0.001

Finally, the same outcome measures were correlated across environments. Marchette Maze pointing was significantly correlated with Virtual SILCton pointing, 95% CI [0.46, 0.73], as was map reconstruction performance in the two environments, 95% CI [0.10, 0.48] and wayfinding efficiency in these environments, 95% CI [0.19, 0.55].

### Confirmatory factor analyses

**Fig. 2:**
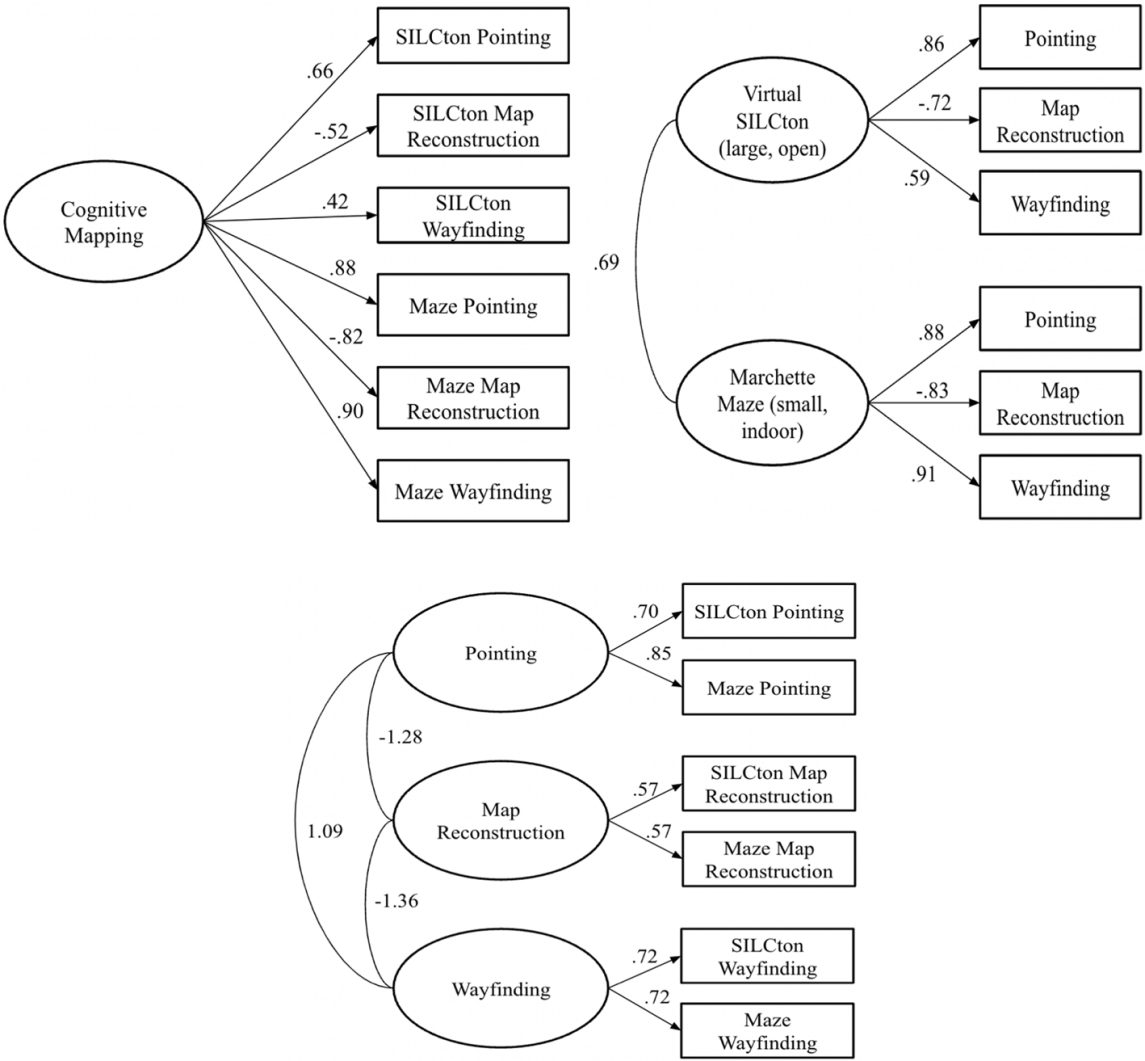
The results of the CFAs for the 1, 2, and 3-factor models

We tested 3 different models in this study. The first model assumed that the six navigation measures (across both Virtual SILCton and Marchette Maze) reflect a single “cognitive mapping” (survey knowledge) factor. The second model assumed that there are two separate factors based on the environment learned, that is, learning large, open, outdoor environments (e.g., Virtual SILCton) and small indoor, grid-like environments (e.g., the Marchette Maze). The third model assumed that there are three separate factors based on the navigation task: wayfinding, pointing, and map reconstruction. Confirmatory Factor Analyses (CFAs) were conducted using the “lavaan” package in R (Rosseel, 2012). Each estimated model, with factor loadings, is shown in Fig. 2. Numbers along the straight arrows are standardized factor loadings; numbers on the curved lines (with no arrows) are the estimated correlations between the latent variables/factors.

Values of fit indices for each factor model are listed in Table 4. For the ${\chi }^{2}$ a significant value means that the model is a poor fit to the data. Since this value becomes less diagnostic with larger sample sizes, we also included the ${\chi }^{2}/df$ statistic, for which a value of less than 2.0 indicates good model fit. The comparative fit index (CFI) assesses how much the examined model fits better than a baseline model and a value greater than 0.95 indicates a good fit (a value of 0.9 to 0.95 indicates a fair fit). Finally, a lower value of the Standardized Root Mean Squared Error of Approximation (RMSEA) indicates better model fit, with a value of 0.05 or lower indicating a good fit and a value between 0.05 and 0.08 indicating a fair fit (Hu & Bentler, 1998).

**Table 4:** Fit indices for the single, two, and three factor models

Model	${\chi }^{2}$	*df*	${\chi }^{2}/df$	CFI	RMSEA
Single factor	46.59***	9	5.18	0.87	0.22
Two-factor	10.52	8	1.32	0.99	0.06
Three-factor	54.98***	8	6.87	0.84	0.26

***p < 0.001

For the one-factor model, the ${\chi }^{2}$ value was significant, and the ${\chi }^{2}$/*df* value was greater than 2.0, indicating a poor fit. Additionally, the CFI value was below the 0.9 threshold (for a fair fit), and the RMSEA was above the indicated threshold of 0.08. Therefore, the one- factor model (i.e., unitary “cognitive mapping” ability) was not considered a good fit to the data.

For the two-factor model, each criterion measure suggested a fair or good model fit. The ${\chi }^{2}$ value was not significant, and ${\chi }^{2}$/*df* was lower than 2.0 (see Table 4). The CFI value was above the 0.95 threshold, indicating good model fit, while the RMSEA fell between the 0.05 to 0.08, indicating a fair model fit. While this model suggests separate factors for the two environments it is also notable that these latent variables were highly correlated (*r* = 0.69) indicating that they also shared considerable variance.

Finally, the three-factor model, proposing separate abilities underlying the three outcome measures was a poor fit to the data. The ${\chi }^{2}$ value was significant, ${\chi }^{2}$/*df* was greater than 2.0, the CFI value was below 0.9, and the RMSEA value was greater than 0.08. Additionally, the correlations between the factors (Wayfinding, Pointing, and Map Reconstruction) exceeded − 1/ + 1, leading to the conclusion that the three-factor model (i.e., that wayfinding, pointing, and map reconstruction abilities reflect different abilities) was not supported by the data.

### Correlations with Sea Hero Quest

Table 5 provides descriptive statistics for Sea Hero Quest wayfinding weighted distance and path integration. Note that due to technical issues, not all participants completed the Sea Hero Quest trials. Sea Hero Quest wayfinding (weighted distance after controlling for Levels 1 and 2) showed good reliability (internal consistency as measured by Omega), but path integration had lower reliability. The Sea Hero Quest measures (wayfinding weighted distance and path integration) were significantly correlated, *r*(67) = − 0.30, *p* = 0.012, 95% CI [− 0.50, − 0.07].

**Table 5:** Descriptive statistics of the self-report measures and Sea Hero Quest (SHQ)

Variable	*N*	*M*	SD	Min	Max	Omega	Skewness	Kurtosis
SHQ Weighted Distance	69	6.44	0.86	4.53	9.42	0.84	0.62	0.84
SHQ Path Integration	69	12.29	1.82	8.00	15.00	0.50	− 0.30	− 0.88
SBSOD	88	4.17	1.08	2.00	6.33	0.92	0.07	− 0.71
Spatial Anxiety	88	2.61	0.74	1.23	4.08	0.92	0.22	− 1.07
Video Game Experience	79	3.28	1.16	1.00	5.00	0.91	− 0.30	− 1.24

In Sea Hero Quest, 23 participants were missing 1 trial in the wayfinding task due to an error with trial data being truncated. To compute descriptives for these subjects, any missing value was replaced with the average weighted distance across all subjects for that trial before being averaged

Table 6 provides the correlations of these measures with the measures of learning Virtual SILCton and the Marchette Maze. Sea Hero Quest wayfinding (weighted distance after controlling for levels 1 and 2) was not significantly correlated with any of the Virtual SILCton or Marchette Maze measures. Path integration ability was significantly correlated only with onsite pointing in the Marchette Maze, 95% CI [− 0.46, − 0.02], but not with the SILCton or the other Maze measures. Even these significant correlations were small in magnitude, suggesting that the Sea Hero Quest measures reflect different abilities to measures of spatial learning.

**Table 6:** Correlations between the desktop environments, self-report, and Sea Hero Quest measures

Variable	SILCton onsite pointing	SILCton map reconstruction	SILCton Wayfinding efficiency	Maze onsite pointing	Maze map reconstruction	Maze Wayfinding efficiency
Sea Hero Quest Weighted Distance	0.13	− 0.08	0.22	0.06	− 0.06	0.15
Sea Hero Quest Path Integration	− 0.22	0.11	− 0.23	− 0.26*	0.11	− 0.15
SBSOD	− 0.31**	0.21	− 0.25*	− 0.18	0.11	− 0.15
Spatial Anxiety	0.32**	− 0.02	0.22*	0.19	− 0.07	0.24*
Video Game Experience	− 0.25*	0.23*	− 0.33**	− 0.45***	0.32**	− 0.44***

*p < 0.05, **p < 0.01, ***p < 0.001

### Self-report measures and their relation to objective measures

SBSOD and spatial anxiety were significantly negatively correlated, *r*(86) = − 0.43, *p* < 0.001, 95% CI [− 0.58, − 0.24] as is typical. Table 5 provides descriptive statistics for SBSOD, spatial anxiety, and video game experience, and Table 6 provides the correlations of these measures with the measures of learning for Virtual SILCton and the Marchette Maze. SBSOD was significantly correlated with onsite pointing, 95% CI [− 0.49, − 0.11], and wayfinding efficiency, 95% CI [− 0.44, − 0.04] for SILCton, but not with map reconstruction for this environment. SBSOD was not significantly correlated with any of the Marchette Maze measures. A similar pattern emerged for spatial anxiety, which was significantly correlated with onsite pointing, 95% CI [0.11, 0.49] and wayfinding efficiency, 95% CI [0.01, 0.41], but not with map reconstruction for SILCton. For the Marchette Maze tasks, spatial anxiety was significantly correlated only with wayfinding efficiency, 95% CI [0.03, 0.42]. In sum, self-reported sense of direction and spatial anxiety had relatively weak correlations with the objective measures, with only some of these reaching statistical significance.

Video game experience was significantly correlated with all environment measures including SILCton onsite pointing, 95% CI [− 0.45, − 0.03], map reconstruction, 95% CI [0.01, 0.43], and wayfinding efficiency, 95% CI [− 0.52, − 0.12], and Marchette Maze onsite pointing, 95% CI [− 0.61, − 0.25], map reconstruction, 95% CI [0.10, 0.50], and wayfinding efficiency, 95% CI [− 0.60, − 0.24]. However, all correlations between environment measures were still significant even after controlling for video game experience (see Table S4 in Supplementary Materials).

## Discussion

We examined the ability to learn two different desktop virtual environments, which simulated an outdoor irregular environment and an indoor maze-like environment, respectively. In each environment, people learned the locations of landmarks via one or more guided routes, and were then tasked with pointing between landmarks, reconstructing a map of the environments, and finding the shortest route between landmarks. We found that performance on all outcome measures were significantly correlated, regardless of the task or environment. We addressed two main questions using confirmatory factor analysis.

1. Does learning of *different environment types* reflect the same or different abilities?
2. Given the *same environment*, do different measures of environment layout (e.g., pointing ability vs. map reconstruction vs. wayfinding efficiency) measure the same or different abilities to learn the layout of an environment?

First, we found somewhat separable factors for the ability to learn large, outdoor, open environments compared to smaller, indoor, grid-like ones for the same outcome measures. However, the latent variables underlying learning of small indoor grid environments and larger outdoor environments were highly correlated (0.69), suggesting that learning the layout of these diverse environments depends on a common navigation ability. Researchers have examined spatial learning in a variety of environment types, some of which are intermediate between the two different environments in our study (see Table 1). Our results suggest that although environmental structure plays a role, after removing variance due to this factor, studies conducted in different contexts measure a common navigation ability so we can generalize across contexts to a large extent. However, they also argue for developing standardized measures, such as SILCton and Sea Hero Quest, which can be used across laboratories, so that researchers in different locations and contexts can be confident that they are measuring the same abilities.

Second, we found no evidence for different abilities associated with different outcome measures, namely, pointing, efficient wayfinding, and map reconstruction. One explanation of these results is that performance on these outcome measures reflects a common type of spatial representation, i.e., survey knowledge with different levels of ability reflecting different accuracy and precision of that knowledge (Peer et al., 2021, 2024). An alternative view is that they reflect different representations (i.e., survey vs. graph knowledge) (Chrastil & Warren, 2015; Muryy & Glennerster, 2021; Warren, 2019) but that acquisition of both types of knowledge reflects the same ability. While the present research does not argue strongly for one of these accounts, it provides no evidence of different abilities underlying the acquisition of survey and graph knowledge (Peer et al., 2021). While some previous studies did not show strong correlations between wayfinding and pointing, in one case the dissociation was found in a non-Euclidean space (Muryy & Glennerster, 2021), and in the other case one of the measures had a floor effect so that it did not discriminate medium from low ability (He et al., 2023).

Regarding the partial dissociation between navigation ability measured in simulated indoor and outdoor environments, we cannot definitively conclude exactly which characteristics of the environments are driving this dissociation. The environments differed in many characteristics, such as size, openness, availability of distal landmarks, environment regularity (only right angles vs. irregular paths). Any one, or a combination of, these characteristics could contribute to differences in performance. Previous research has found that factors such as openness and availability of distal cues affect both how easily an environment is learned, and the type of spatial knowledge that is constructed and indicates that for environments with the same topographical structure, learning of more closed, maze-like environments is subject to greater individual differences (Peer et al., 2024). Our environments also varied in whether people took one route or had to integrate two routes, so they also differed in the need to integrate separate spaces, which Weisberg and Newcombe (2016) have argued is a somewhat separable ability from ability to learn a single space. These considerations argue for further research to identify which aspects of environments and learning experiences account for partial dissociations. For example, one way that future research could address this limitation is by keeping the learning method (a route integration paradigm or single route learning) consistent across the two types of environments.

Moreover, because we examined learning of only one example of each environment, we do not know to what extent these differences are due to different *types* of environments as opposed to different *specific* environments. This should be addressed in future research by examining learning of more than one example of each type of environment. As a step in this direction, Boone et al. (2019) found high correlations (0.70 or higher) between measures of learning two different maze-like environments, which were controlled for complexity (their configurations were mirror images of each other and differed only in surface features such as wall color).

Ability to learn the configuration of an environment from guided routes (the focus of this study) is just one scenario of navigation, raising questions about how this is related to other measures of navigation ability (learning via free exploration, map-based navigation, etc.). As a first step to addressing this question, we examined how our environmental learning tasks relate to map-based navigation and path integration tasks using Sea Hero Quest (Coutrot et al., 2018; Spiers et al., 2021). In general, correlations with the Sea Hero Quest tasks were small and non-significant. There are notable differences between these tasks and the environmental learning paradigm that we focused on here. Environmental learning relies on direct, repeated exposure to each environment to establish long-term memories, whereas Sea Hero Quest relies on working memory of locations on a map before being placed in the environment to navigate to those locations. Additionally, environmental learning involves learning one environment, whereas in Sea Hero Quest the environment changes with each level (trial). As our best-fitting CFA indicated a dissociation between navigating in different environment structures (cf. Barhorst-Cates et al., 2021; Coutrot et al., 2022), it is likely that the Sea Hero Quest environment (a rural environment simulating travel on a boat through irregular waterways) also plays a role in explaining why Sea Hero Quest did not correlate with our measures. Other differences include the display (a desktop computer versus a phone) and gamification (in the case of Sea Hero Quest). Finally, the two Sea Hero Quest tasks themselves were not highly correlated (as also found by Garg et al., 2024), and the reliability of the path integration variable was relatively low.

In general, these results argue for the need to broaden the study of navigation abilities beyond learning of spatial layout, and suggest there may not be one “navigation ability” underlying the various tasks that researchers are currently using to study individual differences in navigation. In addition to environment learning and Sea Hero Quest, these include the ability to find a hidden goal, for example in virtual simulations such as the Morris water maze (e.g., Padilla, et al., 2017) and Star maze (Rondi-Reig et al., 2006), and faceted navigation tasks (e.g., Iaria et al., 2003, 2007; Malanchini et al., 2020; van der Ham et al., 2020), which examine more outcome measures, including landmark and route knowledge in addition to the outcome measures studied here.

Our study partially replicated previous studies regarding the relation between objective and self-report measures of navigation ability and anxiety (Hegarty et al., 2022; Lawton, 1994). Sense of direction was significantly associated with performance in Virtual SILCton pointing, as in previous studies (Weisberg & Newcombe, 2016; Weisberg et al., 2014) but not with wayfinding. Correlations of self-report measures with efficiency in the Marchette maze were somewhat lower than previously reported (Boone et al., 2018; 2019). In general, these results are consistent with previous research indicating weak to moderate relations between self-reports and measures of environmental learning, although weaker than found in many studies, even though self-reports were obtained at the same time as our objective measures and might be expected to be more correlated with these measures. While self-report measures are useful, they cannot substitute for objective measures, supporting the need to develop robust standardized objective tests of navigation.

We found a positive association between performance in each desktop environment and video game experience, raising questions about the extent to which navigation in desktop VR reflects interface facility rather than navigation (see also Hegarty et al., 2022; Yavuz et al., 2024). However, the associations between the environment measures remained significant even after controlling for video game experience, indicating that our tasks are measuring navigation ability, and not just interface facility. Moreover, other studies have shown that navigation in VR environments is correlated with real-world navigation ability (Claessen et al., 2016; Coutrot et al., 2018, 2019; Lader et al., 2024; van der Ham et al., 2015), indicating that using VR environments to study navigation has predictive validity. A main advantage of using desktop VR to measure navigation ability is that we can create standardized tasks, so that labs and researchers all over the world can be confident that they are studying the same abilities. While the types of environments studied here may not be relevant in all cultures, it is easy to create other types of environments in desktop VR that may be more suitable. However, previous research *has* shown a distinction between learning spatial layout via direct experience (locomotion) and via visual media (Hegarty et al., 2006), so it is important to continue to examine relations between desktop VR tasks and measures of navigation in real environments.

## Conclusion

The study of navigation ability is relatively recent and has been conducted in different environments and with different tasks and measures. To develop a consensus on what constitutes navigation ability, and how to measure it, we need to understand the relations between these disparate measures. This study provides a first step in this undertaking. Specifically, it shows that whereas there is some dissociation between ability to learn the layout of irregular outdoor environments and more regular indoor environments, the high correlations between the two learning factors suggests that they depend on a common ability to learn spatial layout. Moreover, while different types of environments might bias the formation of graph-like representations versus cognitive maps, and measures of learning may vary in how much they draw on these representations, there is no evidence that different measures of configural knowledge draw on different abilities. However, our research suggests that other navigation tasks, such as map-based navigation and path integration (as measured by Sea Hero Quest), may rely on aspects of individual differences in navigation that are not captured by studies of environmental learning. Our research demonstrates the importance and necessity of relating paradigms and research approaches across laboratories and contexts to build a consensus on the nature of navigation ability and how it can be measured.

## Supplementary Information

Supplementary file 1.

## Data Availability

The datasets used and analyzed during the current study are available from the corresponding author on reasonable request.

## Abbreviations

CFA: Confirmatory factor analysis
CFI: Comparative fit index
RMSEA: Root mean squared error of approximation
SBSOD: Santa Barbara sense of direction
SHQ: Sea Hero Quest
SILCton: Spatial intelligence learning center test of navigation
VR: Virtual reality

## Footnotes

1 We note that in similar studies (e.g., Chrastil & Warren, 2015; Gagnon et al., 2018; Muryy & Glennerster, 2021; Peer et al., 2024) people learned by freely exploring an environment; these studies are beyond the scope of the current research because they do not control the learning experience and depend on exploration ability.

2 With the exception of the SBSOD, spatial anxiety, and the video game questionnaires, the self-report questionnaires were included for the purpose of a separate study and will be reported elsewhere. However, in general the remaining questionnaires were not significantly related to the variables of interest in this manuscript.

3 For a subset of the participants, the order was reversed for the navigation paradigms (i.e., Sea Hero Quest followed by the Marchette Maze) due to scheduling conflicts, where the lab cell phone was not available for use in the expected timeframe for those participants. However, subjects that had the order reversed did not demonstrate significantly different performance than those with the original task order, so we included them in analyses.
