# Supplementary material for: Learning the layout of different environments: common or dissociated abilities?
Source: Cogn Res Princ Implic. 2025 Feb 21;10:6. doi: 10.1186/s41235-025-00618-5 (PMC11845639; doi:10.1186/s41235-025-00618-5)
Supplement: Supplementary file 1 [file 41235_2025_618_MOESM1_ESM.docx]

**Supplementary Materials**

**Figure S1**

*Example Screenshots of the Virtual SILCton and Marchette Maze Tasks.*

*
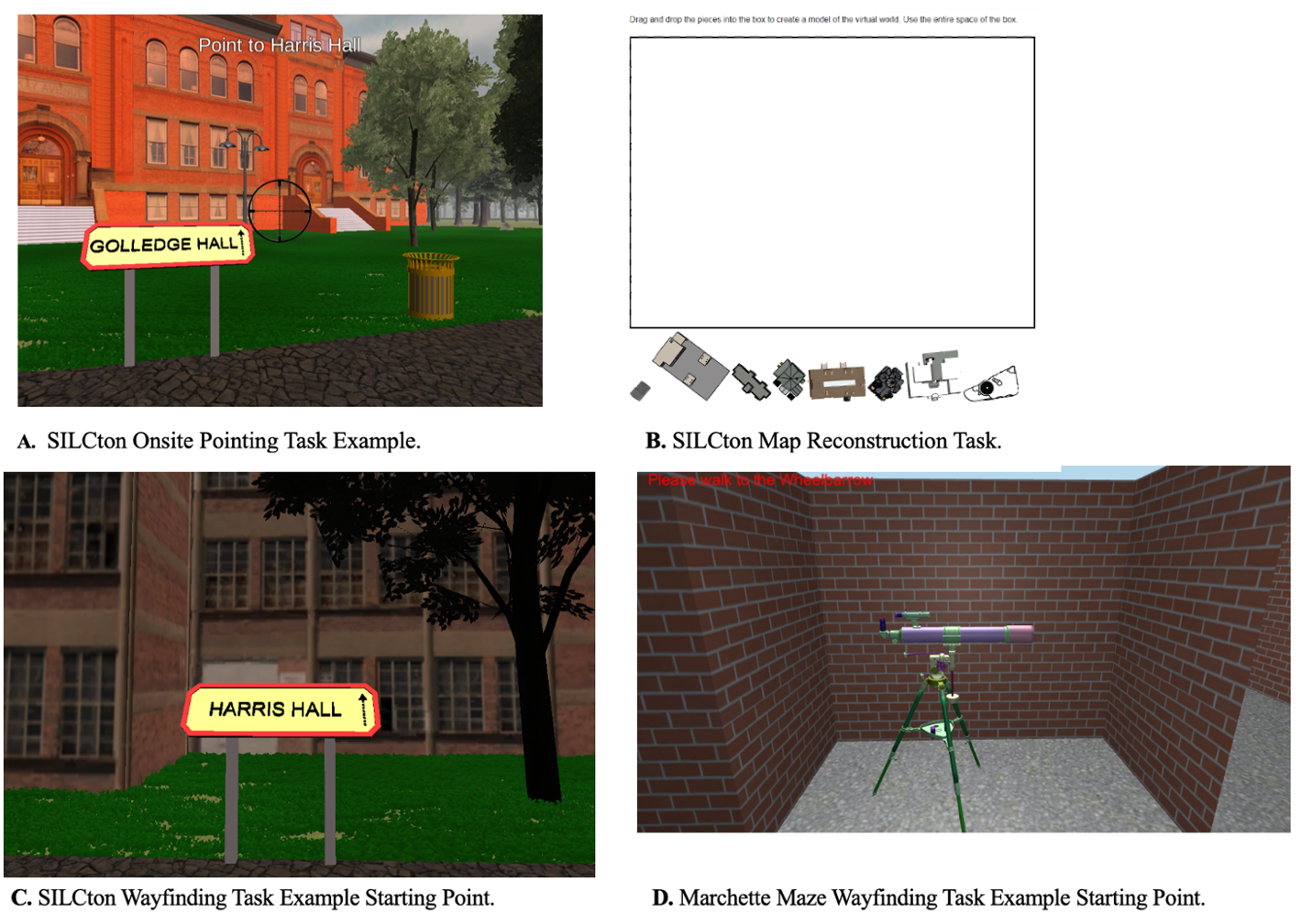
*

*Note:* The Marchette Maze onsite pointing task looks identical to Figure S1D, with the exception of the instructions at the top of the screen stating to point to a target object. The Marchette Maze map reconstruction task is set up in the same way as Figure S1C, except the objects are squares with names and hovering over each square with the mouse provides a picture of the object.

**Video Game Experience Questionnaire**

The following consists of several questions about your general video game experience. *[Scores are averaged across the 4 responses]*

1. How much video game experience do you have?

a. A great deal (5)

b. A lot (4)

c. A moderate amount (3)

d. A little (2)

e. None at all (1)

2. How well do you perform in video games?

a. At expert level (5)

b. Very well (4)

c. Moderately well (3)

d. Not very skilled (2)

e. No skill (1)

3. Reflect on your video game experience in the period of your life that you are/were most active in playing video games. What was the average number of hours per week that you spent on playing video games?

a. 6+ hours/week (5)

b. 4-6 hours/week (4)

c. 2-4 hours/week (3)

d. 1-2 hours/week (2)

e. <1 hour/week (1)

4. How long is/was the period that you are/were the most active in playing video games?

a. >2 years (5)

b. 1-2 years (4)

c. 6-12 months (3)

d. 1-6 months (2)

e. <1 month (1)

| **Table S1**  *Correlations Between the Navigation Measures in the Virtual SILCton and Marchette Maze Tasks, When Assessing Within- and Between-Route Onsite Pointing in SILCton.* | | | | | | |
| --- | --- | --- | --- | --- | --- | --- |
| *Variable* | *1* | *2* | *3* | *4* | *5* | *6* |
| 1. SILCton Within-Route Onsite Pointing | - |  |  |  |  |  |
| 2. SILCton Between-Route Onsite Pointing | .77*** | - |  |  |  |  |
| 3. SILCton Map Reconstruction | -.61*** | -.59*** | - |  |  |  |
| 4. SILCton Wayfinding Efficiency | .49*** | .47*** | -.50*** | - |  |  |
| 5. Maze Onsite Pointing | .60*** | .57*** | -.43*** | .35*** | - |  |
| 6. Maze Map Reconstruction | -.43*** | -.45*** | .30** | -.24* | -.43*** | - |
| 7.Maze Wayfinding Efficiency | .50*** | .55*** | -.45*** | .38*** | .80*** | -.77*** |
| **p*<.05, ***p*<.01, ****p*<.001. | | | | | | |

**Table S2**

*Fit indices for the single, two, and three factor models, when breaking up Virtual SILCton into within- and between-route onsite pointing.*

| Model | $\chi^{2}$ | *df* | $\chi^{2}/df$ | CFI | RMSEA |
| --- | --- | --- | --- | --- | --- |
| Single factor | 91.89*** | 14 | 6.56 | .79 | .25 |
| Two-factor | 16.72 | 13 | 1.29 | .99 | .06 |
| Three-factor | 90.64*** | 13 | 6.97 | .79 | .26 |

****p<*.001.

**Figure S2**

*The results of the CFAs for the 1, 2, and 3-factor models, when breaking up Virtual SILCton into within- and between-route onsite pointing.*

**
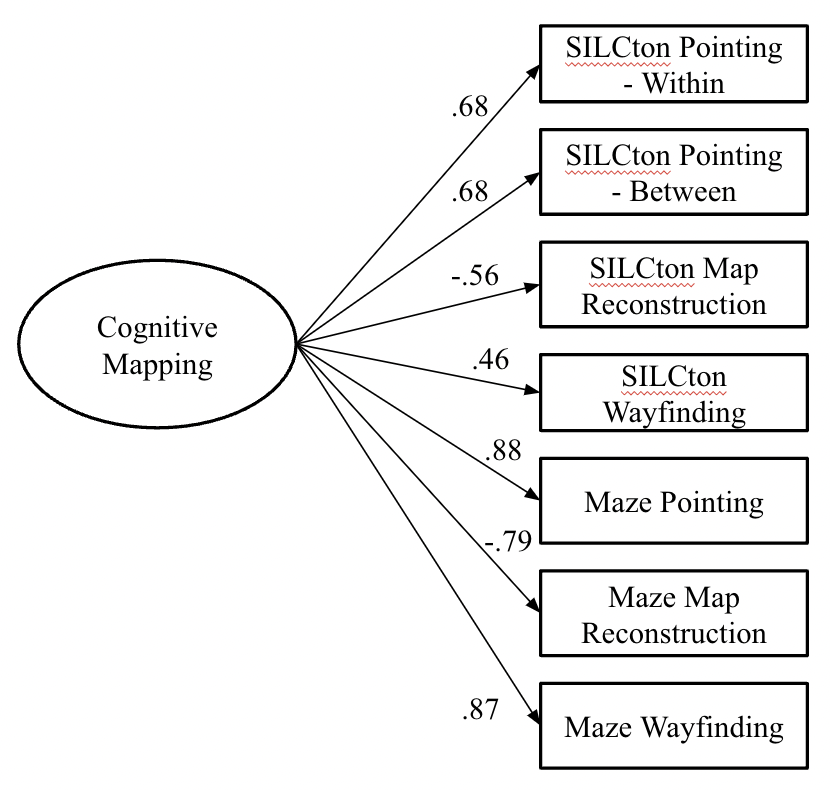

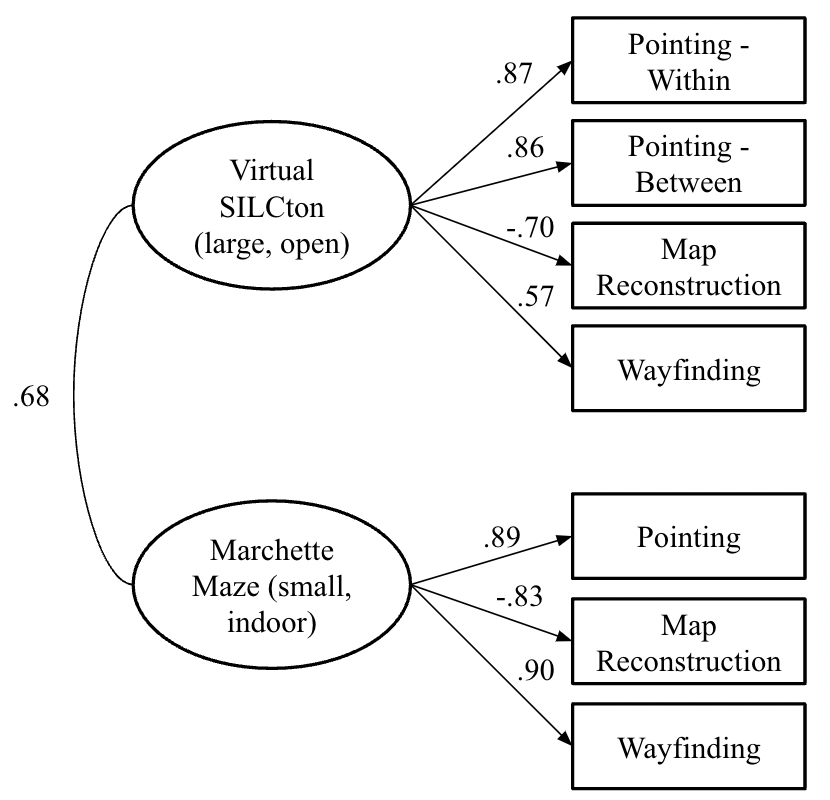

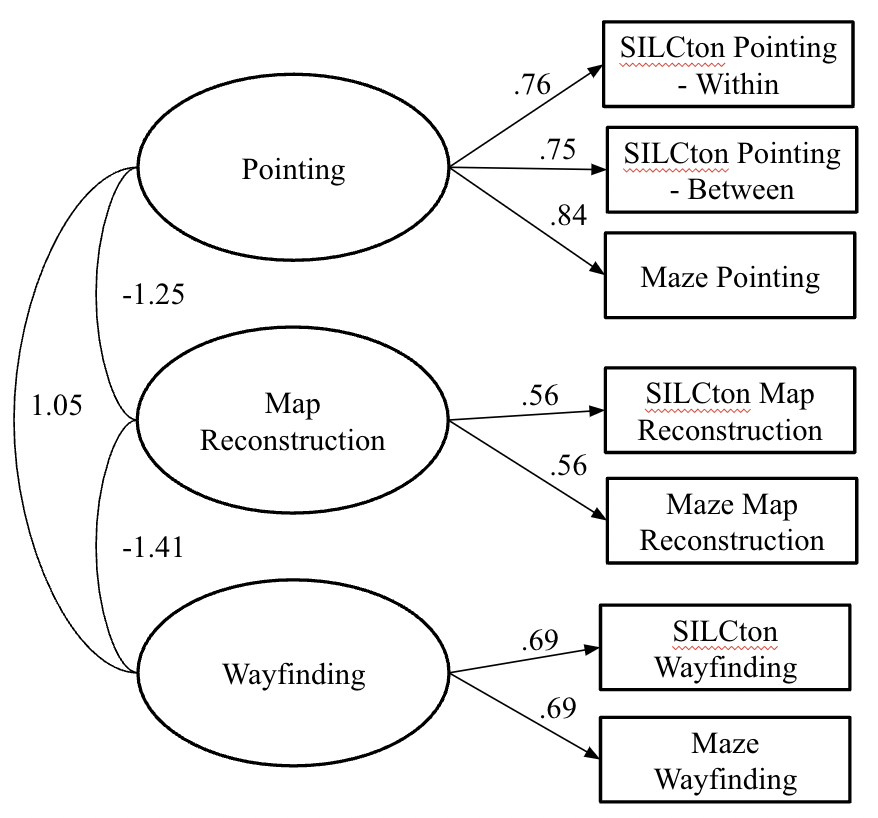
**

**Table S3**

| *Sample Descriptives by Sex Using Welch’s Two Sample t-tests for Unequal Variance* | | | | | | | |
| --- | --- | --- | --- | --- | --- | --- | --- |
|  | Males (*N* = 36) | |  | Females (*N =* 52) | |  | |
|  | *M* | *SD* |  | *M* | *SD* | *t*-test | Cohen’s d |
| SILCton Onsite Pointing | 27.55 | 16.85 |  | 35.19 | 13.10 | -2.28* | -0.51 |
| SILCton Map Reconstruction | 0.74 | 0.26 |  | 0.64 | 0.25 | 1.75* | 0.38 |
| SILCton Wayfinding Efficiency | 1.14 | 0.10 |  | 1.18 | 0.10 | -1.54 | -0.34 |
| Maze Onsite Pointing | 36.00 | 18.93 |  | 47.01 | 17.23 | -2.78** | -0.61 |
| Maze Map Reconstruction | 0.62 | 0.35 |  | 0.52 | 0.30 | 1.41 | 0.31 |
| Maze Wayfinding  Efficiency | 1.53 | 0.42 |  | 1.75 | 0.32 | -2.61** | -0.58 |
| **p*<.05, ** *p*<.01. | | | | | | | |

| **Table S4**  *Correlations Between the Navigation Measures in the Virtual SILCton and Marchette Maze Tasks After Controlling for Video Game Experience (N = 79).* | | | | | |
| --- | --- | --- | --- | --- | --- |
| *Variable* | *1* | *2* | *3* | *4* | *5* |
| 1. SILCton Onsite Pointing | - |  |  |  |  |
| 2. SILCton Map Reconstruction | -.63*** | - |  |  |  |
| 3. SILCton Wayfinding Efficiency | .55*** | -.48*** | - |  |  |
| 4. Maze Onsite Pointing | .58*** | -.40*** | .36** | - |  |
| 5. Maze Map Reconstruction | -.44*** | .26* | -.28* | -.70*** | - |
| 6. Maze Wayfinding Efficiency | .53*** | -.40*** | .38*** | .75*** | -.75*** |
| **p<.05, **p<.01, ***p<.001.* | | | | | |
